# Periodontal Status amongst Substance Abusers in Indian Population

**DOI:** 10.5402/2012/460856

**Published:** 2012-04-17

**Authors:** Shantipriya Reddy, Sanjay Kaul, Chaitali Agrawal, M. G. S. Prasad, Jaya Agnihotri, Nirjhar Bhowmik, D. Amudha, Soumya Kambali

**Affiliations:** Department of Periodontics, Dr. Syamala Reddy Dental College and Research Center, SGR Institutions, Bangalore 560037, India

## Abstract

*Background*. In India there have been limited number of studies on periodontal status among drug addicts, and thus this study aims to assess the Oral hygiene and periodontal status in substance abusers and compare it with non-substance abusers. *Methods*. A comparative study was conducted to assess the periodontal status in substance abusers. Non-substance abusers were procured from the general population of Bangalore. From the control group 250 non-substance abusers were age and sex matched with the study population of substance abusers. The oral hygiene and periodontal condition of all subjects was assessed using Oral hygiene index- simplified (OHI-S), Russell's periodontal indices and Gingival bleeding index. *Results*. The mean of OHI-S and Periodontal Index (Russell's Index) scores were higher (2.70 and 3.68, resp.) in substance abusers than the control group (2.45 and 2.59, resp.). The mean Gingival bleeding score was lower (9.69) in substance abusers than the control group (22.7) and found to be statistically significant. A positive correlation found between OHI-S and Russell's periodontal index whereas negative correlation was found between OHI-S and Gingival bleeding in substance abusers. *Conclusions*. Though the oral hygiene was fair, more periodontal destruction and less of gingival bleeding were observed in substance abusers as compared to control group.

## 1. Introduction

Drug abuse and narcotic addiction are acknowledged problems all over the world having both social and medical implications. They affect a wide range of the population from all socioeconomic classes, and both genders are equally affected.

The health consequences of drug abuse are serious, and the oral health is negatively affected in any society where drug dependency is widespread. This is most likely because of the physical and emotional instability of the addict along with lack of concern for oral health. The prevalence of dental caries and periodontal diseases has been reported to be higher among drug abusers than the rest of the general population.

### 1.1. Alcohol and Periodontal Disease

 Several plausible biological explanations exist for a detrimental effect of alcohol on the periodontitis risk. Alcohol impairs neutrophil function and increases monocyte production of inflammatory cytokines such as tumour necrosis factor alpha (TNF *α*), interleukins 1 and 6, in the gingival crevice contributing to bacterial overgrowth and increased bacterial penetration that may lead to periodontal inflammation. And lastly, alcohol may have a direct toxic effect on periodontal tissue similar to other tissues of the oropharynx.

### 1.2. Cocaine Abuse and Periodontal Disease

 The vasoconstrictive effects of the drug resulted in loss of attachment in the local area and severe recession of the associated buccal periodontal tissue.

### 1.3. Nicotine and Cannabis Abuse and Periodontal Disease

The literature has identified smoker's keratosis or pigmentation changes, oral cancer, and a predisposition to periodontal disease; acute necrotizing gingivitis, candidiasis and xerostomia as oral effects of nicotine abuse. Although bleeding is a sign of periodontal inflammation, in nicotine abuse vasoconstriction in oral tissues leads to reduced bleeding on probing, giving a false clinical indication. Tobacco smoking is a recognized behavioural risk factor for periodontal disease, and cannabis smoking may contribute to periodontal disease in a similar way. Thus in the present study an attempt is made to study the effect of substance abuse on periodontal health in Indian population.

## 2. Materials and Methods

### 2.1. Study Populations and Subjects

After surveying for the deaddiction centres in Bangalore city, Approximately Nineteen deaddiction centres were shortlisted. Out of these seven, were selected randomly by a lottery method. The seven deaddiction centres and general camps were visited from April 2009 to July 2010. The Study population consisted of 500 individuals, in which the study group comprised 250 individuals reporting to the outpatient department of various deaddiction centres in Bangalore city. Subjects were identified as substance abusers according to the International Classification of Diseases 10 (ICD 10) [1] and Diagnostic and Statistical Manual of Mental Disorders (DSM IV) [2]; the Diagnostic and Statistical Manual of Mental Disorders (DSM) is published by the American Psychiatric Association which provides diagnostic criteria for mental disorders. Control group comprised 250 individuals who were nonsubstance abusers, selected from the general population.

Random sample of study group ranged in age from 18 to 64 years and included patients reporting for the first time in OPD of deaddiction centres, who were cooperative for the study. Patients having prior history of periodontal treatment and drug detoxification, having a known history of any underlying systemic disease, and also having a history of intravenous drug abuse were excluded.

Control group consisted of 250 subjects, who did not fulfill the criteria (ICD 10 and DSM IV classification of Substance abusers), were considered as non-substance abusers. From the control group, 250 non-substance abusers were age and sex matched with the study population of 250 substance abusers who were selected from the general population attending routine health check-up camps. The subjects ranged in age from 18 to 64 years and who were cooperative with the study were included. Subjects who were having prior history of periodontal treatment and having a known history of any underlying systemic disease were excluded.

 Ethical clearance was obtained from the concerned authorities of selected deaddiction centres in Bangalore. After obtaining the informed consent, the details of the patient including age, gender, and socioeconomic status were recorded with the help of structured questionnaire. In the substance abuser group various substances consumed were noted and all the patients were screened and further categorized as follows: S.G-1: alcohol abusers, S.G-2: tobacco smoking abusers, S.G-3: cannabis abusers, S.G-4: alcohol + tobacco smoking abusers, S.G-5: alcohol + tobacco chewing abusers, S.G-6: alcohol + tobacco smoking + tobacco chewing abusers, S.G-7: alcohol + tobacco smoking + cannabis abusers, and S.G-8: alcohol/tobacco smoking/tobacco chewing + others. Others which included various combinations of multidrug users like, cannabis, opioids, benzodiazepines, inhalants, heroin, cocaine, methamphetamine, and other solvents.

### 2.2. Oral Examination

The examination (Type-2) was performed using mouth mirror and explorer in the natural light. Patients who are substance abusers and non-substance abusers were subjected to an oral hygiene and periodontal examination which included, oral hygiene Index-Simplified (OHI-S) [3] by Greene and Vermillion 1964, Periodontal Index (Russell's Periodontal Index 1956) [3], and Gingival Bleeding Index [4, 5] to evaluate the periodontal status and compare the results with each other.

### 2.3. Statistical Analysis

The following methods of statistical analysis were used in this study. Proportions were compared using Chi-square (*χ* ^2^) test of significance while one-way analysis of variance was used to test the difference between groups. Student's *t-*test was used to determine whether there was a statistical difference between the two groups for the parameters measured. Data analysis was carried out using Statistical Package for Social Science (SPSS version 10.5).

## 3. Results

### 3.1. Sociodemographic Data

Based on age, the study and control group were further divided into various subgroups. The majority of patients selected for the study belonged to the age group of 30–39 years with the mean age of 37.02 years for the study population and 37.08 years for the control group.

The study group consisted of 250 substance abusers who comprised 230 males and 20 females, and the control group also comprised 250 non-substance abusers which had 227 males and 23 females.

Among all the different types of substance abuse, the substance abuse of alcohol + tobacco smoking comprised maximum number of individuals in the study group, that is, 92 subjects (36.8%) aged between 40 and 49 years in both genders which comprised about 37.0% males and 35.0% females from that group. The substance abuse of cannabis comprised a smaller number of individuals, that is, 6 subjects (2.8%).

### 3.2. Clinical Data

#### 3.2.1. Comparison of (OHI-S) Index in Study and Control Group

The mean value of OHI-S Index in the study group (2.70 ± 0.82 SD) was higher than in the control group (2.45 ± 0.84 SD). This difference was found to be statistically significant (*P* = 0.001), but the oral hygiene scores remained fair (score: 1.3 to 3.0) in both groups (Table 1). 

The mean difference of OHI-S score among males between the study and the control group was statistically significant (*P* = 0.001), but among females difference was not statistically significant (*P* = 0.758).

On comparison of mean OHI-S score between study and control group by age, there was a statistically significant difference between the age groups of <20, 20–29, and 30–39 years with the *P* values of (0.012), (0.035), and (0.010). Moreover, the study also demonstrated poor oral hygiene scores in both study and control groups with advancing age (score: 3.1 to 6.0) (Table 2). 

#### 3.2.2. Comparison of (OHI-S) Index within Substance Abusers

Within the subgroups of substance abusers, the mean OHI-S was maximum (2.96 ± 0.78) for alcohol + tobacco smoking + cannabis and minimum (2.27 ± 0.54) for tobacco smoking (Table 3). However, the difference in OHI-S score among various subgroups was not statistically significant (*P* = 0.106).

#### 3.2.3. Comparison of Periodontal Status (Russell's Periodontal Index) in Study and Control Group

The mean value of Periodontal Index (Russell's Periodontal Index) in the study group (3.68 ± 1.40 SD) was higher than in the control group (2.59 ± 0.81 SD). This difference was found to be highly statistically significant (*P* < 0.001) (Table 1).

The mean Russell's Periodontal Index score of males and females in study group was higher compared to that in control, group and the difference was statistically significant (*P* = 0.000, *P* = 0.007).

In our study we observed poor periodontal status in substance abusers compared to the control group in all age groups with the results being highly statistically significant except in age groups of <20 years and ≥60 years where results were just statistically significant (Table 2).

#### 3.2.4. Comparison of Periodontal Status (Russell's Periodontal Index) within Substance Abusers

Within the subgroups of substance abusers, the mean Russell's Periodontal Index was a maximum of 4.009 ± 1.57 for alcohol + tobacco smoking and a minimum of 3.13 ± 1.20 for alcohol/tobacco smoking/tobacco chewing + others (Table 3). However, the mean difference of Russell's Periodontal Index among various subgroups was not statistically significant with the *P* value of (0.052).

#### 3.2.5. Comparison of Gingival Bleeding in Study and Control Group

The mean value of gingival bleeding in study group (9.69 ± 7.20 SD) was lower than in the control group (22.7 ± 5.13 SD). This difference was found to be highly statistically significant (*P* < 0.001) (Table 1). The mean difference of gingival bleeding among males and females between study and control groups was highly statistically significant (*P* < 0.001) and (*P* < 0.001) respectively.

In our study we observed less gingival bleeding in substance abusers compared to control groups in the age group of 20–29 years, 30–39 years, 40–49 years, and 50–59 years with the results being highly statistically significant (*P* < 0.001) except in the age group of ≥60 years where the results were just statistically significant (*P* = 0.001) (Table 2).

#### 3.2.6. Comparison of Gingival Bleeding within Substance Abusers

Within the subgroups of substance abusers, the mean score of gingival bleeding was a maximum of 14.36 for alcohol and a minimum of 5.56 for tobacco smoking that showed statistically highly significant difference (*P* < 0.001) (Table 3).

#### 3.2.7. Correlation of OHI-S with Russell's Index and Gingival Bleeding Index (Table 4)

There was strong positive correlation (*r* = 0.721) between OHI-S and Russell's Index in the control group and moderate positive correlation (*r* = 0.479) between OHI-S and Russell's Index in the study group. Also there was weak positive correlation (*r* = 0.371) between OHI-S and Gingival Bleeding Index in the control group, and weak negative correlation (*r* = −0.200) between OHI-S and Gingival Bleeding Index in the study group. 

## 4. Discussion

Periodontal disease is one of the most common chronic inflammatory diseases. Though the aetiology of this disease is multifactorial, dental biofilm is considered as the primary etiologic factor. The risk factors are part of “causal chain” and are directly related to disease occurrence. Amongst the risk factors, modifiable risk factors include tobacco smoking, systemic diseases, and other tooth related factors, which modify the host protective mechanism and influence the progression and manifestation of periodontal disease.

 Our study demonstrated that among all the different types of substance abuse, alcohol + tobacco smoking comprised a maximum number in the study groups. This observation is in accordance with the study conducted by Rooban et al. [6], and this could be due to availability and economic reasons.

The majority of the patients in the present study belonged to the age group of 30–39 years with the mean age of 37.02 years in both groups. This observation was also supported by other studies (Brijender et al. [7], Du et al. [8], and Jagadeesan et al. [9]).

 Since the female deaddiction centres were scarce, a few female substance abusers were enrolled. From a total of 250 substance abusers, 230 were males and 20 were females. In contrary, the study conducted by Shimazaki et al. [10] and Ogawa et al. [11] demonstrated a higher number of females with substance abuse. This could be because even today, in India, female drug abusers are very few and not yet open about their disease due to social stigma.

 The mean OHI-S Index score was 2.70 in substance abusers which is comparable to the other studies by Angelillo et al. [12], Shapiro et al., [13], Silverstein [14], Rosenstein [15], and Zahrani [16] who reported mean OHI-S values of 1.71, 2.67, 2.61, 2.2, and 2.42, respectively. The mean OHI-S score was significantly higher in the study group than the control group (*P* = 0.001) especially among the male groups between the age groups of <20, 20–29, and 30–39 years.

 The mean Russell's Periodontal Index score amongst substance abusers was 3.68, whereas other studies by Angelillo et al. [12], Shapiro et al. [13], Picozzi et al. [17], and Rosenstein [15] reported a mean Periodontal Index of 1.37, 2.2, 2.8–3.0, and 2.3, respectively. This difference may be explained by educational and sociodemographic background and ethnic and racial differences. The mean Periodontal index score (Russell's Periodontal Index) was higher in study group compared to control group and this difference was found to be highly statistically significant (*P* < 0.001). One of the reasons of poor OHI-S score and higher Periodontal Index score in substance abusers could be due to poor dental education and awareness as well as lack of motivation.

The mean Periodontal Index scores were higher in both genders and age subgroups in the study group and we also observed greater mean Russell's score in advanced years as compared to younger age group in substance abusers. This is supported by the observations made by the expert group of “WHO” that the destruction of periodontium followed a linear progression from adolescence to old age [9].

Furthermore, our results showed strong correlation between OHI-S and Russell's Index (*r* = 0.721) in control group which suggests that when normal population is considered, the severity of periodontitis strongly correlates with poor oral hygiene. Whereas within the study group, although the mean value of Russell's Index is higher as compared to the control group, the severity of periodontitis has only moderate correlation with oral hygiene (*r* = 0.479). This suggests that, other than neglect of oral health care, there are direct and indirect effects of substance abuse, which are responsible for more severe periodontitis within the substance abusers.

Also the mean Gingival Bleeding Index score was 9.69 in study group compared to 22.7 in control group implying reduced Gingival Bleeding Index scores. Similar findings have been reported in other studies [18]. On further analysis among the categories of substance abusers, maximum gingival bleeding scores were observed in alcoholic group and the least in tobacco smoking group. Furthermore, when OHI-S was correlated with gingival bleeding, our study revealed a positive correlation (*r* = 0.371) in the control group whereas a negative correlation (*r* = −0.200) in substance abusers. This is in accordance with the studies done by Liede et al. [19], Pauletto et al. [20], Bergström and Boström [21], Amarasena et al. [18].

The reason for this negative correlation could be that tobacco smoke induces a vasoconstriction of the gingival vasculature, thereby impairing the gingival blood flow. This in turn might suppress the normal gingival inflammatory response to bacterial plaque and consequently conceal the actual levels of gingival inflammation in smokers. Whereas, alcohol has toxic effect on the liver and as a result the prothrombin production, vitamin K activity, and clotting mechanism may be disrupted. Hemorrhage may occur and this may lead to exaggerated gingival response and bleeding with slightest provocation in alcoholics. Overall less Gingival Bleeding Index scores were observed in the study group probably because most of the categories had tobacco smoking along with other substances.

## 5. Conclusions

Within the limits of this study it can be concluded that even though oral hygiene was fair, more of periodontal destruction, gingival recession, missing teeth, and less of gingival bleeding were observed in substance abusers as compared to control group. All these variables together suggest that oral hygiene and periodontal status of substance abusers was poor as compared to non-substance abusers.

Hence to have clearer interpretation, questionnaire should be revised, and more precise quantification of substance intake should be made. Further studies preferably prospective studies are required to determine the biologic influence of substance abuse on periodontal disease.

## Figures and Tables

**Table 1:** Comparison of OHI-S score, Russell's Periodontal Index and gingival bleeding score between study and control group.

		*N*	Mean	SD	*P* value
OHI-S Index	Control	250	2.455	0.8446	0.001*
	Substance abusers	250	2.704	0.8228	
Russell's Index	Control	250	2.590	0.8167	<0.001**
	Substance abusers	250	3.683	1.4022	
Bleeding on probing	Control	250	22.70	5.139	<0.001**
	Substance abusers	250	9.69	7.209	

**level of statistical significance of less than  .001 with respect to Bleeding index and Russel's periodontal index, *the statistical significance of equal to  .001 with respect to OHI-S in relation to substance abuse and clinical parameters governing periodontal disease.

**Table 2:** Comparison of mean OHI-S score, Russell's Periodontal Index and gingival bleeding score between study and control group by age.

Age		*N*	Mean OHI-S score	Mean Russell's Index	Mean bleeding on probing
<20 yrs	Control	7	0.914	1.486	16.29
	Substance abusers	9	1.789	2.256	13.44
20–29 yrs	Control	60	2.010	2.043	21.68
	Substance abusers	54	2.306	2.831	14.20
30–39 yrs	Control	91	2.431	2.443	23.60
	Substance abusers	86	2.752	3.479	8.07
40–49 yrs	Control	58	2.757	2.993	23.62
	Substance abusers	71	2.928	4.007	8.89
50–59 yrs	Control	24	3.063	3.450	22.54
	Substance abusers	23	3.113	5.270	8.00
≥60 yrs	Control	10	3.220	3.580	20.10
	Substance abusers	7	2.743	6.086	3.71

**Table 3:** Comparison of mean OHI-S score, Russell's Periodontal Index and gingival bleeding score between study and control group within substance abusers.

Substance abusers (subgroups)	*N*	Mean OHI-S score	Mean Russell's Index	Mean bleeding on probing
Alcohol	44	2.664	3.664	14.36
Tobacco smoking	9	2.278	3.978	5.56
Cannabis	7	2.514	3.186	14.00
Alcohol + tobacco smoking	92	2.793	4.009	7.82
Alcohol + tobacco chewing	30	2.937	3.547	11.13
Alcohol + T. smoking + T. chewing	17	2.718	3.429	7.41
Alcohol + T. smoking + cannabis	10	2.960	3.950	9.20
Alcohol/T. smoking/T. chewing + others	41	2.434	3.132	9.07

Total	250	2.704	3.683	9.69

**Table 4:** Correlation between OHI-S score with Russell's Index and Gingival Bleeding Index.

Group		Correlation between OHI-S score verses	
		Russell's Index	Gingival Bleeding Index
Control	Correlation coefficient	0.721	0.371
	*P* value	<0.001	<0.001
	*N*	250	250

Substance abusers	Correlation Coefficient	0.479	−0.200
	*P* value	<0.001	0.001
	*N*	250	250
